# Nikil Mukerji, Adriano Mannino: Covid-19: Was in der Krise zählt. Über Philosophie in Echtzeit: Stuttgart: Reclam 2020, ISBN 978-3-15-014053-6, 120 Seiten, 6 €

**DOI:** 10.1007/s42048-020-00079-z

**Published:** 2020-10-07

**Authors:** Tim Kraft

**Affiliations:** Institut für Philosophie, Universität Regensburg, 93040 Regensburg, Deutschland

Akademische Philosophinnen haben sich spätestens seit Beginn des *shutdown* Mitte März in großer Zahl zur Covid-19-Pandemie geäußert (vgl. o.N. 2020, Rabe 2020). Nikil Mukerji und Adriano Mannino (M&M) sind jedoch die ersten, die dies nicht in Blogbeiträgen oder in der Tagespresse tun, sondern in Buchform. Ihr Buch ist mit programmatischen Überlegungen zu einer Philosophie in Echtzeit verbunden und verspricht eine normative Einbettung in eine Katastrophenethik. Eine der Stärken des Buches von M&M ist daher der Anspruch, Aktualität mit einem programmatisch-theoretischen Rahmen zu verbinden. Das Buch, das mittlerweile die vierte Auflage gesehen hat und auf mehreren Bestseller- und Auswahllisten platziert war, wurde laut Eigenauskunft „während einer Aprilwoche“ (120) niedergeschrieben. Diese Besprechung dagegen wurde im August verfasst, wodurch sie in Pandemie-Zeiten schon zu einem Rückblick wird. Da M&M sich jedoch nicht auf Kommentare zu tagesaktuellen Ereignissen beschränken, ist eine Besprechung nicht obsolet: „Philosophie in Echtzeit“, so der Untertitel, muss nicht wiederum ausschließlich in Echtzeit kommentiert werden. Ob sich die versprochene Philosophie in Echtzeit bewährt hat, kann sich gerade im Rückspiegel am besten zeigen.

Beginnen will ich mit einer kurzen Zusammenfassung der Hauptüberlegungen des Buchs, die vor allem um verschiedene Aspekte des *shutdown *kreisen:

1. *Was ist Philosophie in Echtzeit?* Philosophie in Echtzeit, d. h. Philosophieren unter Zeitdruck über ein Ereignis, während es sich entwickelt und unser Wissensstand darüber sich laufend verändert, ist möglich und nötig. Philosophinnen sollten mit ihrer ethischen Beurteilung nicht abwarten, bis das zu bewertende Handeln in der Vergangenheit liegt und abgeschlossen ist, da die Bewertung dann unser Handeln nicht mehr anleiten kann.
2. *Warum fiel die politische Reaktion auf die sich abzeichnende Pandemie so zögerlich aus?* M&M verschonen die deutsche Politik nicht mit Kritik: Deutschland sei ihrer Meinung nach nicht ausreichend auf eine Pandemie vorbereitet gewesen, obwohl früher oder später mit einer Pandemie zu rechnen war. Zusätzlich seien Maßnahmen bis Anfang März verzögert ergriffen und zögerlich umgesetzt worden. Zur Erklärung ziehen M&M allgemeine Denkfehler und kognitive Verzerrungen heran wie das Präventionsparadox, die Unanschaulichkeit exponentiellen Wachstums und den Truthahnfehlschluss.
3. *Wie sollen wir mit Dissens unter Expertinnen umgehen?* Der Umgang mit Experten will allgemein gelernt sein, stellt uns aber unter Zeitdruck und bei ständig aktualisiertem Wissensstand vor besondere Herausforderungen. Wer für welche Frage zuständig ist, und wann sich Wissenschaftlerinnen zu Fragen außerhalb ihrer unmittelbaren Expertise äußern, ist nicht immer leicht zu erkennen. Wie viele vermutlich erst in den letzten Monaten gelernt haben, sind Virologinnen keine Epidemiologinnen und letztere wiederum keine statistischen Modelliererinnen. Damit nicht genug: Sind sich die Expertinnen vielleicht im Großen und Ganzen einig, sind sie es darüber, wann Schulen geschlossen werden sollten und wann wieder geöffnet, und ob eine Maskenempfehlung/-pflicht für den Alltag ein taugliches Mittel ist, um die Pandemie einzudämmen, noch lange nicht. Darüber herrschte auch unter Expertinnen Dissens. Die Diskussion um die Covid-19-Pandemie ist daher ein interessanter Anwendungsfall für die zwei zentralen Expertise-Probleme aus der sozialen Erkenntnistheorie: Wer ist wofür Expertin? Und was tun, wenn sich Expertinnen widersprechen? Eine gängige Überlegung ist hier, dass man der Mehrheit der Expertinnen folgen sollte (vgl. Grundmann 2020). M&M widersprechen: Der potentielle Schaden bei ausbleibenden oder zu zögerlichen Maßnahmen „gebietet es […], hier der Minderheit der Experten zu folgen und nicht der Mehrheit. Diese zunächst paradoxe Schlussfolgerung ist für den Katastrophenkontext charakteristisch“ (57). Wenn, so ihr Beispiel, Ende März eine Expertin eine Alltagsmaskenempfehlung/-pflicht für notwendig zur Eindämmung der Pandemie ansieht, die Mehrheit dies jedoch nicht tut, sollten wir uns aufgrund des Risikos – hier: irreversible Ausbreitung des Coronavirus mit vielen Todesfällen – der Position der *Minder*heit anschließen. M&M vertreten mit ihrem Prinzip der epistemischen Risikoabsicherung eine Version des *precautionary principle*, das erstens auf Dissens zwischen Expertinnen angewendet wird und zweitens epistemisch, nicht nur praktisch zu verstehen ist.
4. *Was zählt in der Krise, was sagt die Katastrophenethik?* Die Frage, was in der Krise zählt, wird von M&M zunächst ausführlich im wörtlichen Sinne diskutiert: Auf welche *Zahlen *– Infizierte? Dunkelziffer? Reproduktionsrate? Letalität? – kommt es bei der Entscheidungsfindung an? Doch diese Frage bereitet die ethische Frage natürlich nur vor: Was zählt, sei „der Schutz menschlichen Lebens“ und zwar „zunächst vor allem“ (73). Ohne einen *shutdown* ließ sich dieses ethische Gebot nicht einhalten, da wir in der Frühphase der Pandemie nur so „Leben retten und Triage-Entscheidungen vermeiden können“ (74). M&M vertreten daher eine Katastrophenethik, die sich von der Normalethik vor allem dadurch abhebe, dass sie „in weit stärkerem Maße als die ‚Normalethik‘ durch eine Orientierung an Handlungskonsequenzen geprägt ist“ und so erlaube, „sich in der Katastrophe […] über normative Grenzen hinwegzusetzen, die im Normalfall gelten“ (76), insbesondere dann, wenn Triage-Entscheidungen notwendig werden sollten.
5. *Was nun?* Dass ein *shutdown* notwendig und geboten war, um Leben zu retten und die Überlastung des Gesundheitssystem abzuwenden, heißt nicht, dass er zeitlich unbefristet und unabhängig von den sozialen und wirtschaftlichen Folgen ethisch akzeptabel wäre. Wie wir aus dem *shutdown* herausfinden, muss „im Rahmen einer umfassenden Abwägung“ erarbeitet werden, so dass „die Kur nicht schlimmer ausfällt als die Krankheit“ (75).^1^ Die beste Strategie im Umgang mit der Pandemie sei *cocooning plus*: Diese Strategie stellt die Mitglieder der Risikogruppe unter besonderen Schutz *und* zielt zugleich auf eine Eindämmung der Ausbreitung von Covid-19 durch Alltagsmaskenpflicht, Nachverfolgung usw. Das Ziel ist hierbei nicht Herdenimmunität. Sollte sich eine Herdenimmunität durch Durchseuchung der Normalgruppe einstellen, wäre das willkommen, aber Herdenimmunität ist weder das Ziel der Strategie, noch ein Mittel zum Schutz der Risikogruppe. Damit entgehen M&M dem möglichen Einwand, dass die Normalgruppe für den Schutz der Risikogruppe instrumentalisiert werde.

Was ist nun von diesen Überlegungen zu halten? Beginnen wir mit dem programmatischen Konzept einer Philosophie in Echtzeit. Wichtig ist es, sich sowohl über die Abgrenzung zur Philosophie der ruhigen Hand als auch über die Aufgabe der Philosophie in Echtzeit Klarheit zu verschaffen. M&M prägen keinen Begriff für diejenige Philosophie, die nicht in Echtzeit geschieht. Ich habe hier „Philosophie der ruhigen Hand“ gewählt, weil mir diese Bezeichnung am besten die Eigenschaft herauszugreifen scheint, die den Unterschied ausmacht. Der springende Punkt ist nicht, dass wir über etwas Nachdenken, während es geschieht – das gilt für weite Teile der Philosophie –, sondern der äußere Zeitdruck, der sich dadurch ergibt, dass es irgendwann zu spät sein wird. Nachdenken über z. B. Massentierhaltung oder Diskriminierung ist keine Philosophie in Echtzeit, obwohl es um etwas geht, das jetzt, also „in Echtzeit“, geschieht. Es gibt zwar insofern einen Zeitdruck als diejenigen, die über Massentierhaltung oder Diskriminierung nachdenken, sich in aller Regel dafür aussprechen, dass die jeweilige Praxis schnell beendet wird, aber das ist kein äußerer Zeitdruck, da weder ein Ende der Diskriminierung noch der Massentierhaltung in absehbarer Zeit unmöglich werden, wenn wir es bis dahin nicht geschafft haben sollten.^2^ M&M sprechen daher auch treffend von einer Philosophie mit einer Deadline.

Soweit zum Begriff der Philosophie in *Echtzeit*, nun aber zur *Philosophie* in Echtzeit und ihrer Aufgabe. Diese Frage ist u. a. deshalb drängend, weil es Beiträge von Nicht-Philosophinnen gibt, die ähnliche Fragen wie M&M beantworten (z. B. Horton 2020, MacKenzie 2020), und auch Beiträge von Philosophinnen, die ganz andere Aspekte behandeln (z. B. Žižek 2020). Noch schlimmer: Es ist bereits in der Tagespresse der Einwand erhoben worden, gerade der Philosophie falle „außer Banalitäten erstaunlich wenig ein“ (Rabe 2020). Deshalb die Nachfrage: Was zeichnet *Philosophie* in Echtzeit aus und wie unterscheidet sich Philosophie in Echtzeit von *Nachdenken* in Echtzeit? M&M deuten eine mögliche Antwort zumindest an:

In der gegenwärtigen Krise hat sich schnell gezeigt, dass der angemessene Umgang mit der pandemischen Katastrophe nicht allein den Virologen und Epidemiologen überlassen werden kann. […] Vernünftige Entscheidungen erfordern zumal in einer hochkomplexen Entscheidungssituation immer auch die Berücksichtigung und Abwägung vieler anderer Aspekte. (15)

Philosophie wirke also der Zergliederung und dem Wegdelegieren der Debatte entgegen, indem sie eine übergeordnete Perspektive einnehme. Das ist wohlfeil, trifft aber auf die folgenden Kapitel mehr schlecht als recht zu. Wenn M&M zum Beispiel eine Erklärung anbieten, warum viele Staaten schlecht auf eine Pandemie vorbereitet waren und nur zögerlich Gegenmaßnahmen eingeleitet haben, wird nur ein Aspekt herangezogen: hier seien typische Denkfehler exemplifiziert worden. Horton (2020) und MacKenzie (2020) dagegen verweisen in ihren Erklärungen auf weitere Aspekte, nämlich untaugliche politisch-institutionelle Strukturen und redliche wissenschaftliche Irrtümer (vor allem hinsichtlich der Frage, wie relevant a‑ und präsymptomatische Infektionen für die Ausbreitung sind). Bei dem im engeren Sinne ethischen Überlegungen verhält es sich ähnlich: Juristische, wirtschaftliche und psychosoziale Aspekte werden von M&M lediglich erwähnt, um dann schnell beiseitegelegt zu werden, da sich die Katastrophenethik gerade dadurch auszeichne, dass bei drohenden Katastrophen möglichst viel dem Schutz des menschlichen Lebens untergeordnet werde (vgl. 73).

Unabhängig vom Programm einer Philosophie in Echtzeit sind jedoch die konkreten ethischen Überlegungen. M&M konzipieren die Katastrophenethik nicht als eine Bereichsethik wie die Tierethik oder Migrationsethik, sondern als eine Ethik, die greift, wenn die Normalethik durch äußere Umstände (partiell) außer Kraft gesetzt ist. Nur deshalb erlaube die Katastrophenethik die „Einschränkung von Rechten“ (75) bzw. gestatte, sich „über normative Grenzen hinwegzusetzen, die im Normalfall gelten“ (76). Um dieses Konzept einer eigenständigen Katastrophenethik besser zu verstehen, müssen wir verstehen, was eine Katastrophe ausmacht, so dass sie die Normalethik außer Kraft setzen kann, und auf welche Weise das Hinwegsetzen über normative Grenzen normativ begründet wird.

Zur ersten Frage äußern sich M&M nur implizit (vgl. 74, 76). Mal klingt es so, als ob die Anzahl der (drohenden) Todesfälle ausschlaggebend dafür sei, ob die Normalethik außer Kraft gesetzt wird, mal so, als ob es der (drohende) Kollaps des Gesundheitssystems (mit dann notwendig werdender Triage) sei. Das sind verschiedene Begründungsformen, da die Katastrophenethik einmal durch einen (drohenden) aggregierten Schaden – hier: Anzahl Todesfälle –, ein anderes Mal durch (drohende) massive individuelle Rechtsverletzungen – hier: Recht auf grundlegende medizinische Versorgung – ausgelöst wird. Die beiden Begründungsformen kommen bei hypothetischen Situationen auch zu unterschiedlichen Ergebnissen: Eine Grippesaison mit 50.000 Toten kann nach dem ersten Kriterium aufgrund der schieren Anzahl der Todesfälle eine Katastrophe sein, ohne nach dem zweiten Kriterium eine Katastrophe zu sein, nämlich solange alle Erkrankten eine angemessene medizinische Versorgung erhalten.^3^

Auch die zweite Frage lassen M&M überraschend weit offen. Ihnen zufolge lasse

sich eine Analogie herstellen. Danach verhält sich der Shutdown zum Staat wie die Triage zur einzelnen Ärztin bzw. zum einzelnen Arzt. (76)

Der Unterschied hinsichtlich der normativen Begründung könnte jedoch kaum größer sein. Bei der ethischen Bewertung eines *shutdown* gibt es ein naheliegendes normatives Kriterium: Ein *shutdown* mit der damit einhergehenden Einschränkung von Rechten ist ethisch geboten, sofern er der Abwendung einer Katastrophe dient und für dieses Ziel tauglich ist (also weder zu wenige noch zu viele Einschränkungen beinhaltet und sie auch nicht unfair auf die Betroffenen verteilt), d. h. der *shutdown* ist instrumentell begründet. Diese Begründung kann jedoch auf das Handeln nach Eintreten der Katastrophe nicht übertragen werden: Während der *shutdown* dazu dient, eine Katastrophe zu verhindern, kann man instrumentell weder die Anwendung von Triage überhaupt noch ihre inhaltliche Ausgestaltung begründen. Der Kollaps des Gesundheitssystems ist selber schon die Katastrophe, und die ethischen Normen für diese Situation bezwecken nicht, die Katastrophe abzuwenden. Während wir während des *shutdown* einen fairen Beitrag zur Verhinderung einer Katastrophe leisten, ist der (hypothetische) Verzicht auf ein Beatmungsgerät kein Mittel zu irgendetwas, was in unserem Interesse läge.

Auch bei der inhaltlichen Ausgestaltung von Triage-Entscheidungen hilft M&Ms Begründung des *shutdown* nicht weiter. Ob Erkrankte nach Prognose, nach Alter oder *ad hoc* abgewiesen werden, ist gemäß dieser normativen Logik gleichermaßen begründet. Wie M&M zumindest andeuten, wollen sie ein nicht näher spezifiziertes Konsequenzen-orientiertes Entscheidungsverfahren als Rückfalloption heranziehen, wenn die Normalethik nicht mehr weiterhilft. Bevor sie es bei solchen Andeutungen belassen, sollten M&M zumindest prüfen, ob sich die inhaltliche Ausgestaltung von Triage-Entscheidungen nicht bereits vor Eintreten der Katastrophe diskutieren lässt. Dabei könnte man auf einen (tatsächlichen oder hypothetischen) *ex ante* Konsens rekurrieren: In einer Situation, in der wir nicht wissen, in welchem Jahr eine Pandemie welcher Virenart ausbricht, hätten wir eventuell zugestimmt, dass bei Knappheit medizinische Ressourcen strikt nach Prognose verteilt werden. Auch Überlegungen zur Fairness kann man hierbei heranziehen: Jeder Mensch solle eine Chance auf eine angemessene Anzahl von Lebensjahren (*fair innings*) haben, so dass Triage-Entscheidungen nach Alter getroffen werden sollten. Schließlich könnten Rechte auf medizinische Versorgung von vorneherein so konzipiert werden, dass sie nicht zwischen Normalethik und Katastrophenethik unterscheiden, etwa indem wir kein unbedingtes Recht auf eine medizinische Versorgung haben, sondern bei Knappheit ein Recht auf faire Berücksichtigung bei Verteilungsentscheidungen.^4^

Insgesamt verfestigt sich der Eindruck, dass die normative Struktur und die Begründung von M&Ms Position dunkel ist. Es bleibt bei dem vagen Versuch, in der Ethik einen Ausnahmezustand einzuführen, der auf für die Betroffenen unvorhersehbare und undurchschaubare Weise aus Erlaubtem Verbotenes und aus Verbotenem Erlaubtes macht. Normenklarheit wird so nicht erreicht, im Gegenteil.

In der Frage, wie mit Expertinnen-Dissens umzugehen ist, entwickeln M&M eine originelle Position. So vertreten sie die These, dass man in der Maskenfrage frühzeitig der Minderheit der Pro-Masken-Expertinnen hätte glauben sollen, d. h. schon während der Frühphase der Pandemie, als andere Maßnahmen im Zentrum der Debatte standen. Die Diskussion um die Alltagsmaskenempfehlung/-pflicht ist dabei ein interessantes, aufgrund möglicher Rückschaufehler aber auch komplexes Beispiel. Grob gesagt haben sich mehrere deutsche Expertinnen (darunter Wieler, Drosten, Schmidt-Chanasit) Ende März gegen eine Alltagsmaskenempfehlung/-pflicht ausgesprochen, während eine kleine Minderheit (vor allem Kekulé) frühzeitig dafür geworben hat. Wie wir alle mittlerweile wissen, hat sich die Minderheitsposition durchgesetzt. Die naheliegenden Fragen sind nun, ob *erstens* die Mehrheitsposition Ende März richtig oder falsch war und es *zweitens* für Nicht-Expertinnen epistemisch rational oder irrational war, die Mehrheitsposition zu übernehmen. Hinsichtlich der ersten Frage wäre zu prüfen, ob vielleicht Masken Ende März kein relevantes Mittel zur Eindämmung der Pandemie waren, später aber schon. Hinsichtlich der zweiten Frage wäre zu prüfen, ob vielleicht bereits Ende März zu erkennen war, dass es keine gefestigte Mehrheitsposition gab.

Bevor ich aber zu diesen Punkten komme, möchte ich M&Ms eigenen Vorschlag prüfen, dass es unter bestimmten Bedingungen epistemisch rational sein kann, der Minderheit der Expertinnen zu folgen. Der Gedanke, als Entscheidungsgrundlage in Risikosituationen vom Schlechtesten auszugehen, ist im deutschsprachigen Raum seit Hans Jonas’ *Das Prinzip Verantwortung *(1979) aus der Risikoethik nicht mehr wegzudenken. Das Originelle an M&Ms Überlegung ist, dass sie den Gedanken auf den Umgang mit Dissens unter Expertinnen anwenden und dabei auf die Frage, was wir *glauben* sollen. Jonas dagegen spricht explizit von einer „*praktischen* Vorschrift“, nämlich der, dass „der Unheilsprophezeihung mehr Gehör zu geben ist als der Heilsprophezeihung“ (1979: 70, alle Hervorhebungen in diesem Absatz von mir). M&M positionieren sich hier anders, auch wenn man den Text genau lesen muss, um herauszufinden, welche Art von Einstellung – Glauben, Akzeptieren, „Gehör geben“, „leiten lassen“ usw. – sie empfehlen: Es gehe darum, „uns für den eigenen *Erkenntnis*gewinn von den Urteilen einer Expertengemeinschaft leiten zu lassen“ (50), „*kognitive* Arbeitsteilung“ ermögliche „einen höchst effizienten, lukrativen *Erkenntnis*weg“ (51), Ziel sei eine Risiko*epistemologie*, die „als Ethik unserer Überzeugungsbildung“ (15) uns beim Umgang mit „Risikoaspekten von sozialen *Erkenntnis*prozessen“ (51) weiterhelfen solle. An anderen Stellen sprechen sie jedoch davon, wann es geboten sei, „der Minderheit der Experten *zu folgen* und nicht der Mehrheit“ (57), wann wir die von ihnen als wirksam erachteten „Maßnahmen *ergreifen*“ sollten (59) und „wie wir vom Standpunkt der *praktischen* Rationalität aus mit Experteneinschätzungen umgehen können“ (60). Mich lassen diese Formulierungen ratlos zurück: auch nach mehrmaligem Lesen weiß ich nicht, ob hier eine entscheidungstheoretische These – als Antwort auf die Frage ‚Wie sollen wir angesichts von Unsicherheit handeln?‘ – nur mehrdeutig formuliert wird oder auch eine genuin epistemische These – als Antwort auf die Frage ‚Was sollen wir glauben?‘ – vertreten wird.

Dem Schillern zwischen praktischer und epistemischer Lesart scheint folgendes Problem zugrunde zu liegen: Zu den Anwendungsbedingungen des *precautionary principle* gehört, dass überhaupt einmal Nichtwissen bzw. Unsicherheit hinsichtlich der potentiellen Konsequenzen einer Handlung besteht. Um also das *precautionary principle* auf die Maskendebatte anwenden zu können, müssten M&M zeigen, dass diese epistemische Bedingung erfüllt ist. Weder ist eine bloße Möglichkeit geeignet, Unsicherheit zu erzeugen, noch bedeutet jeder beliebige Dissens Unsicherheit. Die bloße Möglichkeit, dass Radiowellen feindselige Außerirdische auf die Erde locken, rechtfertigt alleine auch nach dem *precautionary principle* noch nicht den Verzicht auf Radiosendungen. Ebenso ist es noch keine Unsicherheit, die risikotheoretisch den Verzicht auf Kaffee rationalisieren könnte, dass eine isolierte Minderheit gegen die Mehrheitsposition vermutet, dass Kaffeetrinken ein erhöhtes Krebsrisiko bedeute (vgl. u. a. zu diesem Beispiel Shwed & Bearman 2010). Stattdessen wird das Prinzip paradigmatisch dann angewendet, wenn die Expertinnen sich einig sind, dass ein geringes Risiko großer Schäden besteht (z. B. Kernschmelze bei AKWs) oder sie dies nicht sicher ausschließen können (z. B. Folgen grüner Gentechnik). Dissens alleine ist dabei weder notwendig noch hinreichend für die für die Anwendbarkeit des *precautionary principle* relevante Art von Unsicherheit. Hier können nun aber M&M mit ihrer Überlegung der „asymmetrischen Schadenspotentiale“ (57) ansetzen: Nicht jeder Dissens erzeugt Unsicherheit, aber *dieser* Dissens tue es, weil der Schaden, falls wir Masken irrtümlich nicht tragen (exponentielle Ausbreitung des Virus), viel größer ist als der Schaden, falls wir Masken irrtümlich tragen (geringfügige Belastung des Alltagslebens). Wenn das stimmt, hängt die Antwort auf die Frage, ob die *epistemische* Bedingung für die Anwendung des *precautionary principle* erfüllt ist, von einer *praktischen* Frage ab.

Auch wenn sich auf diese Weise das Argument von M&M vielleicht wohlwollend rekonstruieren lässt, stehen ihm doch einige Einwände entgegen. *Erstens* ist ein derart komplexes Argument gar nicht nötig, um zu zeigen, dass Ende März große Unsicherheit hinsichtlich einer Alltagsmaskenempfehlung/-pflicht als Mittel zur Pandemieeindämmung bestand: Die Expertinnen haben selber darauf hingewiesen, dass sich der Wissensstand laufend aktualisiert, und vor allem gab es offenkundig ein Nebeneinander vieler verschiedener Fragen: Schützt die Maske ihre Trägerin oder ihre Kontakte (falls sie infiziert sein sollte)? Würde eine Alltagsmaskenempfehlung/‑pflicht zu Knappheit und Fehlallokation von Masken führen? Wieviel Infektionsschutz bieten selbstgenähte Masken überhaupt? Und vor allem: Ist die Diskussion um Masken eine von wichtigen Fragen wegführende Nebendebatte, weil eine Maskenempfehlung/-pflicht* alleine* in der Frühphase ohnehin nicht ausreichend gewesen wäre? Schließlich ist auch zweifelhaft, ob es sich wirklich um eine Minderheitsposition handelt (worauf M&M überraschenderweise selber hinweisen, vgl. 58): Nimmt man die globalen Expertinnen, insbesondere auch die ostasiatischen, in den Blick, zeigt sich, dass die Pro-Masken-Fraktion keine isolierte Minderheit ist und Länder mit Alltagsmaskenempfehlung/-pflicht Erfolge bei der Eindämmung von Pandemien erzielen konnten.

*Zweitens *lässt sich der Verdacht kaum vermeiden, dass M&M einem Rückschaufehler aufsitzen: Da es *jetzt* rational ist, an die Tauglichkeit von Masken zu glauben, tendieren wir dazu, es *auch in der Vergangenheit* für rational zu halten. Der Eindruck entsteht dadurch, dass M&M bei ihrem Entwurf des *cocooning plus* auf ihr Prinzip der Risikoabsicherung nicht mehr zurückkommen, sondern einfach von dem ihrer Meinung nach am besten begründeten Konzept ausgehen.

*Drittens* ist unklar, ob sich die Mehrheitsposition überhaupt als falsch herausgestellt hat. So gibt Schmidt-Chanasit zwar zu, dass es sich um einen „wunden Punkt“ handle, bestreitet aber, dass die Mehrheitsposition falsch war: Auf den Punkt gebracht ist seine Verteidigung, dass Ende März Chaos ausgebrochen wäre, wenn eine Alltagsmaskenempfehlung/-pflicht ausgesprochen worden wäre (2020: 19:20–21:33). Auch hier greift, so kann man vielleicht zusammenfassen, das Präventionsparadox: Da das Chaos nicht eingetreten ist, übersehen wir, *warum* es nicht eingetreten ist. Zumindest zeigt dies, dass M&M mit ihrer These der asymmetrischen Schadenspotentiale nicht unbedingt den Expertinnen folgen: Diese hätten den potentiellen Schaden einer Ende März ausgesprochenen Alltagsmaskenempfehlung/‑pflicht wohl viel höher angesetzt als M&M. Für ihr Argument müssen M&M daher erwägen, welchen Expertinnen bei der Ermittlung der Schadensbilanz zu folgen ist. Wendet man hier wiederum das Prinzip der Risikoabsicherung an, fällt man wieder zurück auf die Mehrheitsposition: Das von Schmidt-Chanasit befürchtete Chaos einer Alltagsmaskenempfehlung/-pflicht ist größer als der von Kekulé angenommene Zusatznutzen (zusätzlich zum *shutdown*). Wendet man also M&Ms Prinzips der Risikoabsicherung auch auf die Frage an, ob es asymmetrische Schadenspotentiale gebe, gibt es kein stabiles Ergebnis mehr, da auch hierüber Dissens bestand.

*Viertens *ist es zur Zuschreibung von Verantwortung wichtig, den Ort der Risikoabsicherung richtig zu lokalisieren: Wären wir entgegen der damaligen Mehrheitsposition der Expertinnen davon ausgegangen, dass Masken ein taugliches Mittel zur Eindämmung der Pandemie sind, wäre es irreführend gewesen, dies als Ergebnis einer Konsultation *der Expertinnen* zu verkaufen. Das wäre das Ergebnis einer Risikoabwägung gewesen, die uns als Gesellschaft oder den politischen Entscheiderinnen, aber nicht den Expertinnen zuzurechnen wäre. Wäre sogar, wie von manchen befürchtet, die Maskenknappheit im medizinischen Bereich verschärft worden, wäre es unredlich gewesen, sich darauf zu versteifen, man habe doch epistemisch rational und nach bestem Wissen entschieden, oder gar die Verantwortung auf die Expertinnen zu schieben.

M&M haben ein wichtiges, aktuelles, kurzweilig zu lesendes und anregendes Buch geschrieben, das die Relevanz von Philosophie für aktuelle politische und gesellschaftliche Debatten aufzeigt und einen programmatischen Rahmen für philosophische Beiträge „in Echtzeit“ bereitstellt. Auch Rabes Verdacht, dass die Philosophie „außer Banalitäten erstaunlich wenig“ zu sagen habe, vermag das Buch durchaus zu zerstreuen. Dass inhaltlich viele ihrer Punkte ein weiteres Durchdenken verdient haben, werden vermutlich auch M&M so sehen; dies gilt insbesondere für die Begründungsstrategien, die meines Erachtens auch und gerade in der öffentlichen Philosophie nicht zu kurz kommen sollten. Warum der *shutdown *keine Schnapsidee war, kann man auch der Tagespresse entnehmen; welche ethischen und epistemischen Begründungsfiguren dahinterstehen, dagegen nicht. Man kann daher insbesondere eines aus der Lektüre des Buches von M&M mitnehmen: Öffentliche Philosophie, gerade in Echtzeit, ist wichtig, aber auch schwierig. Wenn wir aus der Covid-19-Pandemie auch mitnehmen, dass öffentliche Philosophie nötiger denn je ist, wir aber darüber nachdenken sollten, wie sie gelingen kann, wäre viel gewonnen.

## Footnotes

1 Die Autoren geben außer dem nicht näher erläuterten Verfahren der Abwägung kein ethisches Begründungsverfahren an; vgl. Lübbe 2018 für Hinweise, warum dieser pauschale Verweis aufs Abwägen wenig hilfreich ist.

2 Hinsichtlich der Massentierhaltung sprechen M&M von einer „tierethischen Katastrophe“ (95, vgl. 100). Sie lassen jedoch offen, ob diese Katastrophe ebenfalls Philosophie in Echtzeit erfordert.

3 Dass Katastrophen nicht nur über die Anzahl der Todesfälle definiert werden sollten, wird auch durch Fangs *Wuhan Diary* nahegelegt. Im Eintrag vom 16.2. gibt sie auf die Frage „Wir befinden uns inmitten einer Katastrophe. Was bedeutet das?“ gleich sieben Antworten, darunter „Katastrophe bedeutet, dass du deinen Angehörigen, der schwerkrank ins Krankenhaus eingeliefert wird, beim Abschied am Klinikeingang zum letzten Mal zu Gesicht bekommst“ (2020: 107 f.).

4 Da auch in Normalzeiten medizinische Ressourcen wie transplantierbare Organe verteilt werden müssen, brauchen wir ohnehin eine Normal-Medizinethik, die nicht allen ein Recht auf alles zuspricht.
